# ‘Dirty pigs’ and the xenotransplantation paradox

**DOI:** 10.1136/medhum-2021-012187

**Published:** 2021-10-25

**Authors:** Gill Haddow

**Affiliations:** Science Technology and Innovation Studies, University of Edinburgh, Edinburgh, UK

**Keywords:** sociology, history of medical, Social science

## Abstract

For almost the last 300 years human beings have sought to use organs from non-human animals to repair or replace their own failing organs. This procedure of intraspecies transplant is called xenotransplantation, and despite the continued attempts by researchers, it is yet to be successful. Experiments in xenotransplantation persist, however, partly based on the perceived biological similarities that exist between humans and non-human animals despite the success of xenotransplantation being hampered by the ability of the human body’s immune system to attack and therefore reject foreign material. In this article, I explore the sociocultural reactions to xenotransplantation which demonstrates that it is based on a paradox; although non-human animals and humans are thought to be biologically compatible or *similar*, many assume and emphasise just how *different* we are from non-human animals. These two positions of ‘same but different’ are arguably incompatible. I begin by reviewing social science research that demonstrates, despite some variation, a range of persistent concerns towards xenotransplantation including the consequences for personal identity should a person receive a non-human animal organ. I add to this body of work, findings from a mixed-method study involving focus groups and a representative survey with young adults to show that most people prefer to have their organs replaced by materials from their own body and non-human animals the least. These reactions sit within a broader context of a 'wisdom of repugnance' that is brought into existence when our classifications of what is thought to be natural or not is threatened.

## Introduction

The nose of the bloodhound will be ours and the ears of the snake; ours also will be the navigational abilities of certain flying insects, which use vibrating fibers in place of gyros. We will have the adaptions of the sonar of the bat and the porpoise. The eye of the eagle may present problems, since its function must presumably be combined with normal human appearance; yet the bettering man [sic] would have to guess that superman’s sight will be better than the eagle at any range (Ettinger 1972, 1)

The above quote is taken from the father of cryonics, RCW Ettinger’s book ‘*Man into Superman: The Startling Potential of Human Evolution and How to be a Part of it’.* In it, he predicts a time when organs and body parts taken from non-human animals could be used to replace and enhance the human form. Indeed, the transplantation of non-human animal organs into humans has been regularly attempted, although unsuccessfully, for at least the last 300 years (Cooper 2012; Lu *et al* 2020). Despite the vision of Ettinger and others since him, xenotransplantation, as the procedure of cross-species or interspecies transplantation is referred to, remains stubbornly elusive. The possibility of exploiting the biological similarities between human and non-human animals for future solid organ transplantation as a therapeutic solution (let alone an enhancement option) remains as intractable as the human organ shortage it is proposed to solve.

Unlike the many years of unsuccessful experimentation between cross-species solid organ transplantation, human-to-human organ transplantation is now an established medical therapy for those individuals whose organs are failing. Since 1967, when Christiaan Barnard transplanted the heart of Denise Duvall into the body of grocer Louis Washansky, accounts from organ transplant recipients persist that the transplantation procedure changes not only their bodies but can also alter aspects of their identity, relating to gender, for example (Fulton, Fulton, and Simmons 1987; Sharp 1995; Sharp 2006; Shildrick 2014; Shildrick *et al* 2009; Simmons and Klein 1987). The modification of the recipient’s body (that is, what they are) also alters their subjectivity, and therefore of who they are; this is a persistent phenomenon that has accompanied human organ donation. It is not my intention to challenge whether the narratives are true nor to take sides in the debate. Rather I suggest that these narratives are important and use them as a starting point to ask: ‘If narratives about inter-species solid organ transplantation are said to alter recipient subjectivity, would this also be the case when intra-species procedures such as xenotransplantation, should become successful?’. This is an important question, not least because the supply of human organs for transplantation has never met the global demand for them; this shortage is arguably one of the drivers to those who continue to conduct experimental research with xenotransplantation.

Non-human animals and humans share similar biological and physiological features and it is this similarity that xenotransplantation experimentation for human organ transplantation is premised on. This sits within a wider discourse of non-human and human similarity in which much of human DNA is shared with a variety of distantly related creatures. Indeed, Robert and Baylis (2003) argue that given the evidence that all living things share a common ancestor, there is little (if anything) uniquely human. The emphasis on the similarities between non-human animals and humans has led to the increasing value of non-human animal materiality for human therapy.

This discourse of the value of the shared physiological features between human and non-human is challenged however by an awareness of the rights of non-human animals based on a recognition that they share emotional and cognate abilities similar to human beings. This is partly why the term ‘nonhuman animals’ is used by animal activists and academics in the field of animal studies1 (Francione 2008).

Non-human animals are thought to share some of the social characteristics of human beings, and human beings are said to share some physical characteristics of non-human animals. Yet, these different notions of similarity and difference create a paradox within xenotransplantation. That is, xenotransplantation rests on a boundary that is supposed to exist between non-human animals and humans, which makes non-human and human *the same but different*. Herewith the paradox; on the one hand, xenotransplantation relies on the perceived biological similarity of organs between human and non-human animals and yet on the other, xenotransplantation challenges both the biological and social boundary of difference that makes humans separate and different to non-human animals. Hinterberg draws attention to this anomaly in her analysis of regulatory decision-making whereby ‘regulators and policymakers now find themselves in a curious position. On the one hand, they continue to regulate the estrangement between humans and other animals, but on the other, they support the creation of chimeric life—a form of life that draws into question the very basis of such separations’ (Hinterberger 2020, 1065).

To demonstrate the xenotransplantation paradox (that is, 'nonhuman animals are the same as humans but different') I begin reviewing research on patient, health professional and public attitudes that have consistently demonstrated negativity towards xenotransplantation (Appel III, Alwayn, and Cooper 2000; Bona *et al* 2004; Brown 1999; Brown and Michael 2004; Butler 1998; Canova *et al* 2006; Conesa *et al* 2006; Cook 2013; Fovargue 2007; Hagelin 2004; Lundin 2002; Lundin and Idvall 2003; Lundin and Widner 2000; Macer *et al* 2002; Martinez-Alarcon *et al* 2005; Michael and Brown 2004; Mohacsi *et al* 1995; Mohacsi *et al* 1997; Mohacsi, Thompson, and Quine 1998; Persson *et al* 2003; Rios *et al* 2007; Sanner 2005; Schlitt *et al* 1999). At present, pigs are thought to be an ideal source for transplantation into humans as their internal organs are similar in size to humans but this is not often identified as the organ donor host in previous research. I outline a mixed-model research design I used, combining focus group research with a representative survey of young people in the UK. I explore in the research the imagined preferences for different procedures to replace, repair or regenerate human organs that are failing or failed. For example, if participants are offered a hypothetical choice between human and non-human sources (the latter including smart implantable devices as well as pig organs) what choice would be made and what does such choice tell us about human identity? (Haddow 2020). In this article, I reaffirm the overall aversion for xenotransplantation as well as offering reasons for it that are multivarious relating to (1) The possible unethical treatment and use of non-human animals generally; (2) The physiological, functional and immunological incompatibility between non-human animals and humans; (3) Specifically that pigs are ‘dirty’ provoking negative reactions because of their association with dirt, and finally (4) That xenotransplantation procedures contest the constructed boundaries between what it is to be a non-human animal and human. I conclude with thoughts about ‘yuck’ as defined by the bioethicist as moral wisdom and relate this to the concept of disgust by the anthropologist Douglas (1966) which is understood as ‘the reaction which condemns any object or idea likely to confuse or contradict cherished classifications’ as out of place (Douglas 1966, 36). Taken together, this highlights how the paradox of being ‘the same but different’ lies at the heart of xenotransplantation and the intangibility of how non-human animals and humans relate to one another. It is due to the paradox that possible transgressions of the boundary between species has to be monitored and controlled because it is on closer inspection biologically indeterminate and socially contestable.

## Human organ transplantation and alterations in subjectivity

Since the recipients of cadaver organs, like those with organs from living relatives, often express the sentiment that one can acquire the donor’s emotional, moral, or physical characteristics. Such qualities can be elaborate and imaginative, especially when the donor was an anonymous stranger. Some patients live in fear of the independent or animate qualities of the new organs (Sharp 1995, 372).

### Previous social science research

Clinical organ transplantation between human beings has been successfully taking place for the last 50 years and is no longer the experimental treatment that it once was when the first heart transplant was conducted on 3 December 1967, by the South African heart surgeon, Christiaan Barnard. He did so by removing the heart from 25-year-old Denise Darvall who had died in a car accident and placing it into the body of grocer Louis Washansky. Louis, perhaps like others before him who are the ‘first’ in experimental procedures, died only 18 days later. His death from pneumonia was a result of complications due to suppressing his immune system to stop his body from rejecting Darvall’s heart (Høystad 2007).2 Until advances in immunosuppression were made human organ transplantation procedures remained unsuccessful (and as I discuss below this is a problem that xenotransplantation has yet to overcome). The media storm that ensued after Louis’ death was not because he had died as a result of the experimental procedure that should not have taken place, nor indeed whether in South Africa, the apartheid system would create a situation whereby one section of the population would become a source for another (Bound Alberti 2010). Instead, journalists asked Louis how it felt to have a female heart or one that was not Jewish (Nathoo 2007).

Five years after Louis’ death Fox and Swazey published ‘*The Courage to Fail’* (Fox and J 1974) outlining the social and ethical dilemmas emerging with the emerging practice of clinical human organ transplantation. Fox and Swazey identified an ‘anthropomorphization’ of the donated organ by the recipients which are in some way rehumanised or socialised with the donor’s presence (Fox and Swazey 1992). For example, one organ transplant recipient suggested to them that, ‘I had a strong feeling that I had a part of another man’s body; a man that I didn’t even know’ (1974: 31). Simmons *et al’s* review of several studies in the early 1970s suggested ethnicity, youth and gender were all characteristics thought to have been transferred from the donor (Simmons and Klein 1987). Similar findings were to persist decades later when organ transplant recipients were found to be more welcoming of an organ transplant from a man as better and stronger as opposed to an organ from a female donor (Sanner 2003, 394). Sharp's (1995) ethnography of 26 kidney recipients, suggested the integration of an organ such as a lung could result in a generic ‘transformative experience’. Indeed, in Sharp’s research, she found that ‘some patients live in fear of the imagined independent or animate qualities of the new organs (Sharp 1995, 372). In her book, *‘A Change of Heart: The Extraordinary Story of a Man’s Heart in a Woman’s Body’* lung-and-heart transplant recipient Claire Sylvia (1997)Sylvia and NovacK 1997 details the subjectivity alterations she experienced after receiving an organ from a young male donor she said she knew nothing about. To demonstrate that knowledge of the donor host was not pivotal as the source of identity alterations, the research attempted to match the changes that occurred in the recipient with the unknown characteristics of the donor (Pearsall, Schwartz, and Russek 2002). As a result, Pearsall *et al* argue that despite no knowledge of the donor ‘sensitive’ transplant recipients can experience ‘changes in food, music, art, sexual, recreational and career preferences, as well as specific instances of perceptions of names and sensory experiences related to donor’ (Pearsall, Schwartz, and Russek 2002, 191). Recent research conducted by Shildrick (Shildrick 2010; Shildrick 2015) suggests that few of the 30 heart transplant recipients in her study, were able to view the heart as a ‘transferable and disembodied organ that has shed all vestiges of its prior location…aware that their sutured bodies spoke to a different mode of being-in-the-world’ (Shildrick 2010, 18). What this body of research demonstrates is that organs ‘are always more than mere things’ (Lock 1995) and that although the organ is removed from the human body, it can have a narrative that continues its social life (Parry 2018).

### Pharmaceutical and biological explanations

Some commentators and health professionals doubt the veracity of the narrative of altered subjectivity and point to the effects of immunosuppression to stop the rejection of the transplanted organ. Organ donor recipients must take immunosuppressants to lower their immune response system, but it is also a medication known for side effects such as developing sugar cravings. One of the surgical pioneers of organ transplantation argued that cell migration from the donor to the recipient is an essential part of the organ being accepted into the body and can be found throughout the recipient’s body, with 'both the allograft and recipient become genetic composites' (Starzl *et al* 1993). This idea of the recipient becoming a genetic composition of themselves, and the donor is debated. Although it is acknowledged that the organ donor recipient will be a mix of two different types of DNA, it is argued that the donor DNA remains located at the site of the organ and does not circulate throughout the recipient’s body.

A related idea to organ transplantation resulting in the recipient being a composite of DNA is that organs have a ‘cellular memory’ base, and that cellular memory is the cause of the subjectivity alteration experienced by the recipient. Whether cellular memory, genetic composition or pharmaceutical response, all of these explanations reside in the biomedical realm of knowledge about human bodies and are not adequately addressing why particular *social* characteristics are believed to be transferred. I turn next therefore to show that some individuals believe the social characteristics of non-human animal donors are also thought to create subjectivity alterations through modifying the body via xenotransplantation.

## Xenotransplantation

HG Wells wrote in 1896, ‘*The Island of Doctor Moreau’*, describing how the shipwrecked Prendick discovers an island where non-human animals are being turned human. In a short novel written in 1915, ‘*The Metamorphosis’* by Franz Kafka, the narrative of transformation is reversed when the human protagonist awakes as a monstrous insect. There is fascination with complete transformations from animal to human and with different combinations of humans and animals. For example, from ancient Greek and Egyptian times with sphinxes (human-lion combinations), centaurs (human-horse combinations) and fauns (human-goat) to more present-day modern fictional accounts of werewolves and mermaids. In Greek myth, Icarus flew too high to the sun and melted the glue holding his wings that he made from feathers, plunging him into the sea and causing his death by drowning. Accompanying the fable is an implicit message about 'flying too high' in terms of the limits of emulation and ambition. Whether it is humans fully or partially morphing into non-human animals, or non-human animals turning into human, these are only a few examples of what appears to be a continuing cultural enthralment with the connection humans have with non-human animals.

Xenotransplantation could some day turn the fiction of human and non-human animal hybridity into a reality. Yet for many years, xenotransplantation despite it being heralded as having the potential to address the human organ shortage, the advances in successfully creating a non-human animal and human hybrid remain modest.3 Currently, the only successful procedures of transferring and implanting non-human animal parts into humans are through using decelluralised porcine or bovine material to replace human heart valves. Xenotransplanted organs maintain their non-human animal cellular structure unlike the decelluralised materials used for heart valves, which makes organs liable to attack by the organ recipient’s immune system. Attention is now turning to the success of gene-modification techniques such as CRISPR-Cas9 that could humanise non-human organs working in tandem with immunosuppression. Such a breakthrough was reported in 2016 when a genetically modified pig’s heart was placed inside a baboon’s abdomen, with the baboon surviving for over 900 days (Mohiuddin *et al* 2016).

### Of pigs

Our cultural fascination with interspecies transference and animal-human hybridity turns into repugnance however if people are presented with xenotransplantation as a possibility. A review of different acceptance rates in potential transplant patients and carers (transplant waiting patients/patients who had transplants; dialysis patients and patients with type 1 diabetes; healthcare professionals and members of the public/students) found an agreement to the possibility of xenotransplantation varied greatly from 80% finding it acceptable dropping to 19% depending on how much information was given about how successful the procedure would be (Stadlbauer *et al* 2011). Social and cultural beliefs about the acceptability of xenotransplantation are likely to vary and may be dependent on need, preference, the amount of animal material used as well as where it was going to be implanted. It is possible that those who are more likely to need a xenotransplant would be more likely to be open to receiving one. Although there are no successful examples of solid organ xenotransplantation replacing human organs with animal ones, using decellularised structures such as heart valves are used. Some research with patients who have received small non-cellularised tissues instrumentally viewed the porcine implants with little evidence of concern (Idvall 2006; Lundin 2002; Teran-Escandon *et al* 2005), although Lundin also found anxiety in her research with patients with diabetes transplanted with insulin-producing porcine islet cells (Lundin 1999). One patient with diabetes who had received porcine islets reflected: ‘It feels like something big and meaty. And I am wondering what way it can change me as a person. Yes, not that I'll develop a tail or anything like that—but that something will happen to me all the same’ and ‘Like small piglets …tiny pig cells that I have no control over and that can pump something animal-like into my body’ (Lundin 2002: 337). Recipients implanted with decellularised porcine heart valves also demonstrated similar concerns about the transference of animal qualities (Lundin 1999; Lundin 2002; Lundin and Widner 2000). A patient with Parkinson’s disease reflects on the possibilities of using animal tissues in another study, suggesting that ‘The personality is in the brain. If you add a very small quantity of cells from a pig to an existing brain, that’s OK. But if we are talking about replacing half of the cerebrum, then we would be replacing a large share of the individual’s personality’ (Lundin and Widner 2000, 1175).

### Public attitudes towards xenotransplantation

Many studies have sought to gauge public attitudes towards xenotransplantation. These are often based on attitude scales, and the results paint a relatively similar picture to each other. They show xenotransplantation can be as acceptable as human organ transplantation (80%–90% would accept such a procedure if necessary), but that this support drops significantly if more information about xenotransplantation is given. Or if the given scenario suggests that xenotransplantation would not be as good as a human organ transplant (Bona *et al* 2004; Canova *et al* 2006; Conesa *et al* 2006; Lundin and Idvall 2003; Lundin and Widner 2000; Martinez-Alarcon *et al* 2005; Rios *et al* 2005; Sanner 2006). Concerns found in this research related to disease transmission or a possible transference of genetic material, ethical issues with xenotransplantation practice, as well as fears ‘about the psychological aspects of having an animal organ in the body’ (Stadlbauer et al 2011, 498). Some researchers found more favourable attitudes towards smaller amounts of non-human animal cells and tissues such as porcine heart valves rather than larger organs in the general population (Persson *et al* 2003). Lesser amounts of non-human animal organs are found to be more acceptable especially when it comes to the broader social importance placed on the brain in terms of human identity (Stadlbauer *et al* 2011). The logic presumably is that with decreasing amounts of materiality used from the source, the less likely the risk of contaminating the recipient. It appears that it is not only a case of how much of the human body requires replacement but where the substitutions are made and whether it is of the same material or from a different origin (eg, whether human vs non-human animal).

Focus group research indicates the mixing of non-human animal and human organs produces reactions of disgust from members of the public (Brown 1999; Brown 2009; Brown and Michael 2001; Brown and Michael 2004). For ‘[Fl]esh is something about which we are culturally ambivalent, even when it comes to eating it … [m]oving it about in the fashion of xenotransplantation is hardly likely to be culturally neutral’ (Birke and Michael 1998, 252). Indeed, there is an increasing number of individuals turning to vegan and vegetarian dietary choices on ethical, environmental and health grounds as well as religious objections to the consumption of pork.

## Current study

### Sample

In 2016, a series of four focus groups were carried out, followed by a representative questionnaire-based survey of young adults.4 The focus group study was conducted first for several reasons; mostly to explore views about the acceptability of using human, non-human animal or mechanical devices in such procedures in a deep, interactive and meaningful manner. The focus groups were initially purposively sampled for age, religion, sporting activity and familiarity with technology, and convenience recruitment via known contacts was used; hence (1) The 65 years of age and over, (2) University Competitive Fencers, (3) Computer Gamers and (4) members of a University’s Islamic religion group. Although identification of group members was based on primary characteristics (such as being preinternet citizens in the case of the over 65s or assumed technology embracers such as the Computer Gamers, competitive sports for individuals focused on body work, or known religious views regarding meat in the cases of Islamic religious group) the participants’ identities varied by experiences, demographics and interests. For example, Roy in ‘The 65 Years of Age and Over’ group and a ‘pre-internet’ citizen was a committed vegan which strongly affected his views of xenotransplantation as further discussed below. The focus group data that were generated partially informed the next phase of data collection, which was a series of questions in a survey format to young adults.

### Data collection

The focus group discussions took place in a mutually agreeable location generally lasting an hour and a half with a moderator and facilitator having a role in each of the discussions.5 Areas of discussion began with exploring ideas about the relationship an individual has with the body and was followed by a wide-ranging conversation about human organ transplantation, willingness to accept a xenotransplant, as well as novel technologies such as 3-D bioprinting. In the focus groups permission was sought to record and reassurances about confidentiality were given (pseudonyms are used in the following accounts). Focus groups were transcribed verbatim and the text imported into a computer-aided qualitative data analysis package aiding the management of data (Nvivo V.11). A constant comparative method generating codes from the data, and themes from the inter-relations between codes was used and is an approach loosely informed by Grounded Theory (Charmaz 2006). However, a more abductive approach to thematic generation was taken overall, that is, with a knowledge of previous research and a sensitivity that new and unanticipated data would emerge (Blaikie 2007).

From 11 years to 17 years of age, 1550 young people were targeted in a survey. This age cohort was chosen because they are (1) Possibly more open to technoscientific solutions to organ transplantation given they are internet citizens and (2) Least likely to perceive themselves in need and therefore offer responses unaffected by the possibility of requirement. The overall sample of young people comprised around 300 state secondary schools throughout Scotland, UK. The survey was administered by class teachers, using self-completion online questionnaires in a mixed ability class such as Personal, Health and Social Education. The questions were generated in close collaboration with Ipsos MORI, commissioned to carry out the study and are a large UK market research company (https://www.ipsos.com/ipsos-mori/en-uk). General demographics collected in the survey were age, gender and religion as well as eating preferences such as vegetarianism (eg, was there a connection between vegetarianism and being against xenotransplantation). Views could be captured by asking young people to indicate their most and least preferred options and the following options and short explanations were given and rotated in different orders in the survey:

- An organ is taken from a pig (a statement to describe xenotransplantation);
- A mechanical device that did the work of the organ; (to refer to implantable medical devices);
- A spare organ is taken from someone you knew who was alive (to avoid confusion it was stated that this was a related living organ donation and with an organ that was needed hence ‘spare’);
- An organ grown from your cells in a laboratory (3-D bioprinting);
- An organ is taken from a stranger who has recently died (the current UK system largely based on deceased organ donation).

All the human organ options (3-D bioprinting, living donation and deceased donation) were the favoured options with 3-D bioprinting by far the most preferred (22.3%), as shown in table 1.

**Table 1:** How would you *most* want the organ replaced?

Frequency		Per cent
An organ is taken from a pig	25	1.6
A mechanical device that did the work of the organ	123	7.9
An organ is taken from a stranger who has recently died	179	11.5
A spare organ is taken from someone you knew who was alive	336	21.7
An organ grown from your own cells in a laboratory	345	22.3
Don’t know	407	26.3
Prefer not to say	135	8.7
Total	1550	100.0

The focus groups with ‘Computer Gamers’, ‘University Islamic group’ and ‘The 65 Years of Age and Over’ groups, stated their top preference would be for 3-D bioprinted organs thus confirming an overall preference in the results, for an organ to be created from one’s own body. As Sophia in the ‘Over 65’s’ group suggests ‘I would prefer to have something that is connected in some way to a human being either past or present or manufactured from something in the…Well, just having a connection to a human in some way, even it was made from cells cultured in the lab originally, an imaginary source’.

### 3-D bioprint me

Statements in the open part of the survey supporting 3-D bioprinting ranged from, ‘They (organs) come from me’ emerges, being from ‘my own cells’, ‘it was your own’, ‘part of my body’, ‘part of me’, ‘my own cells in my own life’ and that this was preferred from other human bodies, as it was from ‘my own body and not from someone else’. However, Adila, in the ‘University’s Islamic’ focus group pointed out that should 3-D bioprinting become a possibility in the future, potentially avoiding issues around rejection (as is the case, for example, with human transplantation and xenotransplantation) this would be an expensive first world option:

I think I would, of course, prefer my own stem cell and my reason is like what I pointed out earlier. Sometimes our body rejects a new organ, someone else’s stem cell might have a different reaction, there is a risk, the issue of risk. However, going back to the initial stem cells in, yes, I would prefer that, but on the other hand I think it’s quite an exclusive option because there are many countries, we cannot afford such technology, and we have to depend on a human donor, so it’s great, but it’s very limited in how it reaches up to people and there are a lot of people who are in need of organs, and probably people from first world country could develop this technology. The only thing your own stem cells do for organs that can be in turn donated to people who cannot afford it in third world countries, I think that’s a great option, yeah (Adila).

The human living options were stated as being the preferred option, although deceased organ donation (n=179) is the least popular of all the human options. One reason for ambivalence about deceased organ donation was that the deceased donor was a stranger, for example, ‘I don’t know the person or how they lived their life’, ‘because it seems risky and I wouldn’t know their past’. The other reason for dislike was that the donated organs came from an individual who had died and ‘because they are dead and that’s weird’, ‘I don’t like the thought of someone’s dead organs in me they wouldn’t work’. Those who supported deceased donation did so for the same reason that those against it gave; it was ‘because the person was dead’ and the ‘organs would not go to waste’.

Organs from deceased donors appear more disliked by young men (38%; n=109) than young women (61%; n=69) in the current sample. An explanation or reason for why young male respondents were more likely to dislike the current way that organs are procured from deceased donors is unclear. The next best substitute would be of a known living individual; the quality of being known seems almost as important as being human. A reason for not favouring the current deceased procurement system, considering these future possibilities, is because the donor is both deceased and a stranger. So, to summarise, an organ created from the individual is preferred, a known organ donor is liked, but a deceased stranger’s organ will do. What will not do, however, is using organs from a pig as I turn to next.

### Never xenotransplantation

3-D bioprinting potentially avoids ethical, practical, religious and social challenges posed by xenotransplantation (Brown 1998). The survey results (shown in table 1 above) demonstrate less than 2% of the young adults (n=25) gave xenotransplantation as their preferred option. It was said by a few individuals in the focus groups, to be very similar to consuming meat. In the ‘Computer Gamer’ focus group, the following exchange occurred between Aidan, Oliver and Timothy:

Aidan: Does it make a difference if you can use the rest of the pig for meat?

Oliver: That makes it better. That makes it better.

Aidan: What’s that again, what’s that kind of meat? Sorry.

Oliver: If you get a heart for a transplant…

Timothy: And, bacon.

Oliver: …then you also get bacon, so…

Aidan: To ruin the heart that you just got.

Timothy: To be fed to the person that’s got the heart.

Apart from the apparent jocular nature of the exchange between the male focus group participants, a view is expressed that if a person consumes meat, then this should make the person hold positive opinions about using animals for xenotransplantation. In the ‘65 Years of Age and Over’ focus group Roy, who was a lifelong vegan, expressed a similar view in a far less humorous tone in an exchange with Cameron (who consumed meat):

Roy: I’m a 30 years vegan, so it’s quite clear my decision on that, I think it’s an appalling idea. I think it is again the exploitation of animals and whatever which I think, don’t think we really should be involved in…

Cameron: Not enthusiastic.

Roy: We agree, yes, we have found common ground probably for totally different reasons.

Cameron: Given that I enjoy a bacon sandwich, I think it would be illogical for me to say I wouldn’t take something from a pig, again providing that it is done without cruelty.

Roy: …if you’re prepared to put it down your throat, then why wouldn’t you be prepared to put it in your leg or whatever, your heart?

The challenge from Roy is that those who consume meat should be in favour of xenotransplantation as ‘…if you’re prepared to put it down your throat, then why wouldn’t you be prepared to put it in your leg or whatever, your heart?’. The survey results paint a different story, however. Roughly equal numbers of those who self-identified as vegetarians (46%) and those who consumed meat (48%) said they were against xenotransplantation. It may be that practice of transplanting pigs’ organs is not the equivalent of choosing to consume pork or ham. Through the digestive process, meat consumption will leave the body, whereas xenotransplantation will not and would be a permanent addition. Neither does eating meat instigate a rejection process by the body, whereas a xenotransplanted organ does. In the ‘Competitive Fencer’s’ group, Amy discusses the issue of immunosuppression required for the recipient’s body so as not to attack a non-human animal organ:

Amy: If it was a last resort, I would definitely accept an animal organ. But I would accept a human organ over an animal organ if they were both available. Because even if it was like perfectly functional, the same, but there are risks associated with animals because they are different, physiologically. So, if you get down to like cellular level with all the receptors and everything, it means you have to be on…I know you have to be on immunosuppressants in a human, but you have to be on more, I think, with an animal.

Such discussions echo the challenges of overriding the body’s immune response to attack any organic materiality that is foreign to the recipient’s body.

If lifestyle decisions about meat consumption appear to have little relationship to xenotransplantation, does religious instruction forbidding eating meat, especially pork, play a role in being against xenotransplantation? Some authors have suggested it is acceptable for religions against eating meat, for example, those who identify as Muslim, to accept pigs as organ substitutes (Welin and Sandrin 2006) despite instruction that says otherwise. Fifteen of the 27 respondents who self-identified as Muslim in this survey, were not in favour of using pig organs for xenotransplantation. Some participants chose to identify themselves as Muslim in the open comments section, saying that because the pig was not halal, it was an ‘unclean’ animal. In the following exchange, which occurred in the ‘University’s Islamic’ group between Azzam and Leilah, Azzam is trying to articulate why pork consumption is unacceptable:

Azzam: …For example, like pigs, are seen in Islam as…so if you look like…I'm trying to say…like for example, pigs and stuff like they also like…the reason why they don't…I think the reason is because pigs are like…they play around in the mud and stuff.

Leilah: Lay there in faecal matter.

Azzam: And they also eat their…

Leilah: Faecal.

Azzam: Yes and their own poo, so they’re generally seen…I was thinking of a way not to say that, by the way, if you didn’t get it. So yeah.

Importantly, the association of pigs with dirt was articulated regardless of religious affiliation. The frequency of the comments (such as, ‘It’s yuck, disgusting, gross, unclean’, ‘It’s a farm animal with a very unhealthy diet’ and pigs are ‘disgusting, rank, not natural, grim, vile, rank’) outnumbered any explicit or implicit link to religious affiliation. Not only was the pig thought dirty it could also be a vehicle for diseases, ‘the pig could have had a disease’, ‘because pigs are disease-ridden creatures’, ‘pigs can have some nasty diseases’.

Other comments were made that were not as clearly articulated such as ‘It just feels strange’ and ‘It doesn’t sound right’, ‘it would especially make me feel mentally uncomfortable’, ‘it would creep me out’, ‘it’s not nice to think about’, ‘I don’t want a pig/animal inside me’. Having a pig’s organ would make someone ‘part-pig’ and that the risk of using non-human animal organs was not just about practical or ethical questions but had to do with personal identity issues. Comments included: ‘I would hate to have an organ from an animal, I wouldn’t feel right having a pig’s organ’, ‘I don’t wanna be part pig, cos I would be pig’, ‘I don’t want a pig inside me’, ‘I would feel awkward about having a pig organ’. Very often the reason given in the survey’s comments section for being against xenotransplantation was stated matter-of-factly: *‘It’s a pig’.* This exact phrase that occurred frequently, suggesting a shared understanding of the reason that pigs would not be acceptable, did not require any further elaboration. One last comment worth mentioning is that not everyone thought that pigs’ organs would affect identity, as Adila suggests: ‘I don't think it affects me as a person. I think it…if I needed it, it’s urgent, I might die without it, I think I would take it and it will not affect me as a person, I'm really sure of it. Neither my cognitive ability, my spirituality, my emotions, so…’. This was not a common response, but it does highlight that a person’s need for an organ, even a non-human animal’s one in the case of xenotransplantation, may overcome any social or cultural ambivalence and abhorrence about the source.

## Conclusion and discussion

Repugnance, here as elsewhere, revolts against the excesses of human willfulness, warning us not to transgress what is unspeakably profound. Indeed, in this age in which everything is held to be permissible so long as it is freely done, in which our given human nature no longer commands respect, in which our bodies are regarded as mere instruments of our autonomous rational wills, repugnance may be the only voice left that speaks up to defend the central core of our humanity. Shallow are the souls that have forgotten how to shudder (Kass 1997, 20).

When given a choice responses in the current research suggest a majority view against xenotransplantation and hugely favourable response in favour of 3-D bioprinted ones followed by organs from a known individual and then a deceased stranger. The popularity of 3-D bioprinting and the desire to have personalised organs relate to avoiding concerns about bodily functioning that xenotransplantation raises (reliability and compatibility), avoidable harm to others (including non-human animals) and knowledge about the source of the organ. Although pigs that would be used for xenotransplantation would be clinically and medically grade pathogen-free, responses in the current research suggest the pig is thought to be dirty, physiologically incompatible, and potentially a vehicle of disease. As I have shown, there is little variation in terms of religion or vegetarianism beliefs that helps explain respondents’ negative views of xenotransplantation. Views about xenotransplantation might be therefore closer to understandings about chimaeras and the mixed bodies of species, rather than vegetarianism. The creation of chimaeras and hybrids, for example, is seen as ‘an affront to the hierarchical superiority and separateness of the human species’ despite the practice of breeding animals and hybridised plants (Knoppers and Joly 2007, 284).

Indeed, as shown in the focus groups and survey, were the imagined possibilities of becoming ‘part pig’. Xenotransplantation, as is the case with beliefs about possible gender alteration through human organ donation, potentially threatens an individual’s subjectivity. Previous studies show that proposals to mix non-human animal and human organs produces public reactions of disgust or ‘yuck’ (Brown 1999; Brown 2009; Brown and Michael 2001; Brown and Michael 2004). ‘Yuck’ echoes anthropologist Mary Douglas’ arguments about challenges to the boundaries between species and is linked to ideas about ‘Pollution behaviour’ which is ‘the reaction which condemns any object or idea likely to confuse or contradict cherished classifications’ as out of place (Douglas 1966, 36).

The finding from this research suggests a deeper-seated repugnance expressed as ‘yuck’ due to the perceived challenge to what is considered the natural species’ boundaries. Pollution behaviour is invoked when controversial crossing and blurring of boundaries between bodies and species occurs. Pigs are entities that transgress familiar and taken-for-granted boundaries between species (Alter 2007; Chakrabarty 2003; Robert and Baylis 2003). This suggests that it is important to maintain identity and integrity on two levels. First, the possibility similar to the narration from human organ transplant recipients, that pig organs can alter a person’s subjectivity through the modification and breach of the integrity of the body with an organ from another (once) living being. Second, this breach is not from a human being, and therefore the boundary that separates humans from non-human animals is at risk of transgressing the categories of what is known to be human and what it is to be a non-human animal. Kass (1997) relates ‘yuck’ to a ‘wisdom of repugnance’ that is not just a matter of individual taste but is a powerful way to discuss how reactions to the way that such proposals are challenging what is considered as ‘natural’ as taken up by others in Science and Technology Studies:

The contemporary need for naturalness can be better understood as a response to the fact that technology makes reality more and more makeable and, consequently, more contingent. Advancing technology changes everything that is, into our object of choice…[I]f human nature itself becomes makeable, it can no longer naively be laid down as the norm (Swierstra, Van Est, and Boenink 2009, 274).

The paradox of xenotransplantation is one that simultaneously highlights how deep the need is for a natural boundary to exist between what is human and what is a non-human animal and yet how shallow the socially constructed division between the species is.

## Data Availability

No data are available.

## Notes

1. I prefer to use the term non-human animals to contrast both the similarities and the differences inherent to the xenotransplantation paradox.

2. When Louis died, the heart was removed as it was required by South African police and as Denise had died in a car accident thus ‘the heart…was an important part of two different people with separate histories’ Nathoo (2007). “The Transplanted Heart: Surgery in the 1960s.” *The Heart*. J. Peto. New Haven and London, Yale University Press.

3. Xenotransplantation remains an experimental procedure and has a high failure rate, as was tragically demonstrated in the1980s when Baby Fae died after her failing heart was replaced with one from a baboon, raising questions about the ethics of the procedure, especially whether her parents gave full and informed consent. Kushner, T. and Kushner and Belliotti (1985). *Baby Fae: A Beastly Business. A Question of Give and Take: Improving the Supply of Donor Organs for Transplantation.* B. New, M. Solomon, R. Dingwall and J. McHale. London, King’s Fund Institute Report. 18.

4. The research was part of a larger Wellcome Trust funded project called ‘Animal, Mechanical and Me: The Search for Replacement Hearts 100561/Z/12/Z. NHS ethical approval was gained for all fieldwork (REC 13/SS/0232).

5. My thanks to Dr Tirion Seymour for her help in recruiting and conducting the focus groups.
